# German S3 Guideline Nonrestorative Sleep/Sleep Disorders, chapter “Sleep-Related Breathing Disorders in Adults,” short version: German Sleep Society (Deutsche Gesellschaft für Schlafforschung und Schlafmedizin, DGSM)

**DOI:** 10.1007/s11818-017-0136-2

**Published:** 2017-11-07

**Authors:** Geert Mayer, Michael Arzt, Bert Braumann, Joachim H. Ficker, Ingo Fietze, Helmut Frohnhofen, Wolfgang Galetke, Joachim T. Maurer, Maritta Orth, Thomas Penzel, Hans Pistner, Winfried Randerath, Martin Rösslein, Helmut Sitter, Boris A. Stuck

**Affiliations:** 1 Schwalmstadt-Treysa, Deutschland; 2 Regensburg, Deutschland; 3 Köln, Deutschland; 4 Nürnberg, Deutschland; 5 Berlin, Deutschland; 6 Essen, Deutschland; 7 Mannheim, Deutschland; 8 Erfurt, Deutschland; 9 Solingen, Deutschland; 10 Freiburg, Deutschland; 11 Marburg, Deutschland

## Contents

1.: What’s new?
2.: Classification of sleep-related breathing disorders
2.1: Obstructive sleep apnea
2.2: Central sleep apnea
2.3: Sleep-related hypoventilation/sleep-related hypoxemia
3.: Diagnosis
3.1: Diagnostic recommendations
4.: Obstructive sleep apnea—recommendations
4.1: Positive airway pressure therapies
4.2: Non-positive airway pressure therapies
5.: Perioperative management of patients with obstructive sleep apnea—recommendations
6.: Diagnosis and treatment of central sleep apnea—recommendations
7.: Diagnosis and treatment of sleep-related hypoventilation/hypoxemia—recommendations

## 1. What’s new?

**Fig. 1:**
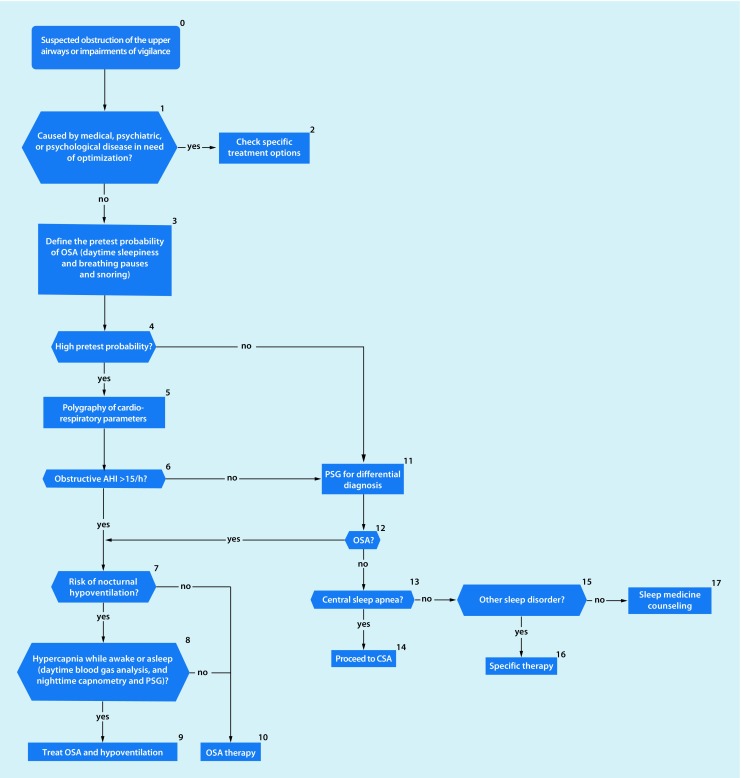
Algorithm for management of patients with suspected obstruction of the upper airways. Following exclusion of medical and psychological diseases requiring optimization and in the presence of a high pretest probability, i. e., daytime sleepiness *plus* breathing pauses *plus *snoring, polygraphy of cardiorespiratory parameters can be a sufficient diagnostic instrument. In the case of a low pretest probability, polysomnography (*PSG*) is performed for differential diagnosis. (*AHI* apnea–hypopnea index,* OSA* obstructive sleep apnea, *CSA* central sleep apnea)

**Fig. 2:**
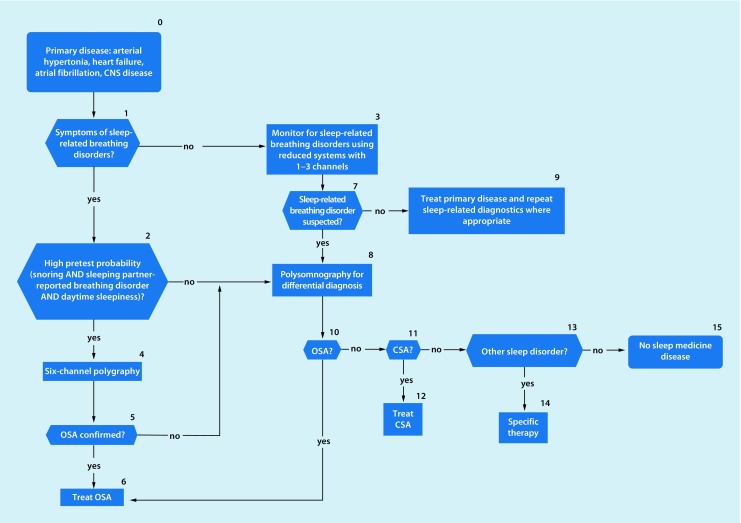
Algorithm for management of patients with cardiovascular diseases. Around 50% of patients with cardiovascular disease are also affected by a sleep-related breathing disorder. Therefore, in asymptomatic cardiovascular patients, monitoring for sleep-related breathing disorders can be performed with simplified systems with 1–3 channels. If symptoms of sleep-related breathing disorders are present, polygraphy or polysomnography is indicated. (*CNS* central nervous system, *OSA* obstructive sleep apnea, *CSA* central sleep apnea)

**Fig. 3:**
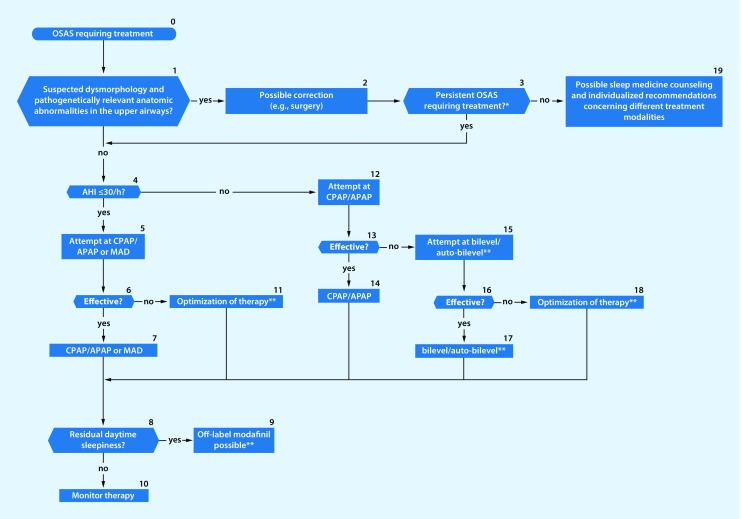
Algorithm for treatment of patients with obstructive sleep apnea. *Patient training, behavioral recommendations, sleep medicine counselling; in overweight patients, weight reduction should be attempted in parallel. **In patients with an apnea–hypopnea index (*AHI*) ≤30/h and lifelong obstructive sleep apnea (*OSA*), positional therapy can be considered if no other therapy is possible or tolerated. Mandibular advancement devices (*MAD*) can also be considered in patients with severe sleep apnea who do not tolerate or refuse continuous positive airway pressure (*CPAP*), or in whom CPAP therapy cannot be used despite utilisation of all support measures. Where positive airway pressure therapies or MAD fail, in the absence of anatomic abnormalities and the presence of an AHI of 15–50/h, neurostimulation of the hypoglossal nerve (NSHG) can be used up to class I obesity, provided there is no concentric obstruction of the airways. (*OSAS* obstructive sleep apnea syndrome, *APAP* automatic CPAP)

**Fig. 4:**
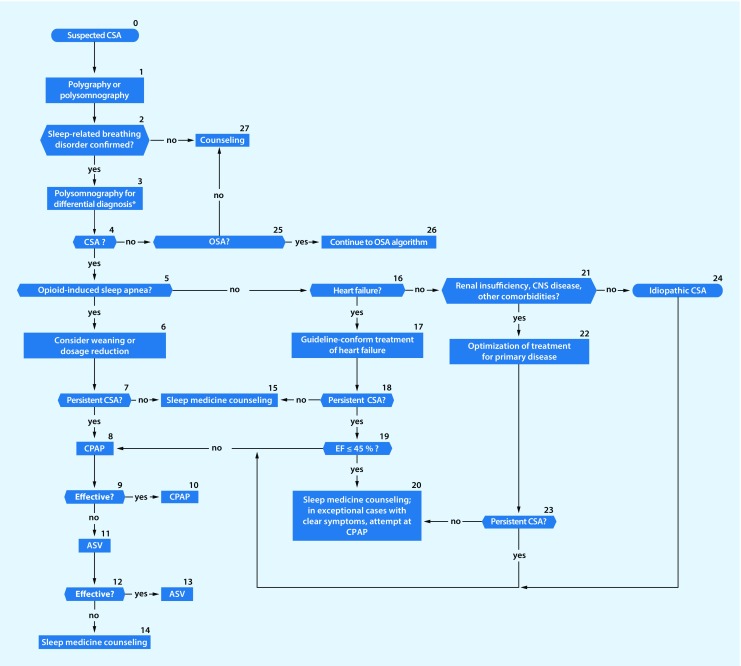
Algorithm for management of patients with suspected central sleep apnea. *Upon unclear findings in polygraphy, polysomnography is performed for differential diagnosis. (*CSA* central sleep apnea, *CPAP* continuous positive airway pressure, ASV adaptive servoventilation,* CNS* central nervous system, *OSA* obstructive sleep apnea, *EF* ejection fraction)

- Within the past 20 years the prevalence of obstructuve sleep apnea (OSA) has increased by 14–55%.
- Sleep-related breathing disorders (also termed sleep-disordered breathing, SBD) are common in patients with heart failure. They are also associated with increased morbidity and mortality in subjectively non-hypersomnic patients.
- There is an association between OSA and malignant diseases.
- Maternal OSA during pregnancy can harm the newborn.
- Untreated sleep apnea increases cognitive decline in patients with dementia.
- Patients with a high probability of a sleep-related breathing disorder and a high accident risk should receive diagnosis and initiation of any required treatment as early as possible.
- Clinical examination should include inspection of the nose, oral cavity, pharynx, and dental status, as well as evaluation of facial skeleton morphology. A cephalometric radiograph may be obtained to establish facial bone morphology.
- The “STOP-BANG” questionnaire has been incorporated into the diagnostic spectrum.
- Polygraphy systems should only be used when there is a high pretest probability of diagnostic confirmation and for determining the severity of a sleep-related breathing disorder.
- In cardiovascular disease patients without typical symptoms of a sleep-related breathing disorder, simplified systems with 1–3 channels can be used.
- Questions addressing OSA should be included in preoperative assessments of case history.
- In case of suspected undiagnosed OSA a diagnostic sleep work-up should be performed; however, the urgency of surgery must be weighed against the necessity/type of sleep medicine diagnostics on an individual basis.
- The technical possibilities for telemonitoring have improved considerably during recent years. Telemedical care of patients with a positive airway pressure device is now technically possible through nightly recording of treatment-relevant parameters.
- Algorithms have been developed for
  - the management of patients with suspected obstruction of the upper airways (Fig. 1)
  - the management of patients with cardiovascular diseases and sleep-related breathing disorders (Fig. 2)
  - the treatment of patients with OSA (Fig. 3)
  - the management of patients with suspected central sleep apnea (Fig. 4)

## 2. Classification

Sleep-related breathing disorders manifest exclusively or primarily during sleep. They disrupt sleep and thus impair its restorative function. Characteristic patterns of disturbed breathing are apneas and hypopneas with or without pharyngeal obstruction, and hypoventilation. Depending on the type of breathing disorder, it may be accompanied by hypoxemia, or cause hypercapnia and acidosis.

The International Classification of Sleep Disorders Version 3 (ICSD-3) distinguishes between five diagnostic categories, the names of which are based on the patterns of disordered breathing during sleep or the underlying pathomechanism (Table 1). Within these five categories, ICSD-3 contains descriptions of 18 diseases.

**Table 1:** Diagnoses of sleep-related breathing disorders according to the International Classification of Sleep Disorders Version 3 (ICSD-3; AASM 2014)

Main category	Subcategory	ICD-10-CM
Obstructive sleep apnea	Obstructive sleep apnea in adults	G47.33
	Obstructive sleep apnea in children	G47.33
Central sleep apnea	Central sleep apnea with Cheyne–Stokes breathing	R06.3
	Central sleep apnea due to a medical cause without Cheyne–Stokes breathing	G47.37
	Central sleep apnea due to high-altitude periodic breathing	G47.32
	Central sleep apnea due to drugs or substances	G47.39
	Primary central sleep apnea	G47.31
	Primary central sleep apnea in children	P28.3
	Primary central sleep apnea in premature infants	P28.4
	Central sleep apnea due to therapy	G47.39
Sleep-related hypoventilation	Obesity hypoventilation syndrome	E66.2
	Congenital central alveolar hypoventilation syndrome	G47.35
	Late-onset central hypoventilation with hypothalamic dysfunction	G47.36
	Idiopathic central alveolar hypoventilation	G47.34
	Sleep-related hypoventilation due to drugs or substances	G47.36
	Sleep-related hypoventilation due to a medical cause	G47.36
Sleep-related hypoxia	Sleep-related hypoxia	G47.36
Isolated symptoms and normal variants	Snoring	R06.83
	Catathrenia	

Early diagnosis and treatment, e. g., of OSA, reduces the risk of accidents and improves quality of life. Currently it is assumed that untreated OSA leads to increased healthcare costs. In contrast, effective treatment of OSA represents a cost-efficient measure in terms of healthcare economics.

### 2.1 Obstructive sleep apnea

OSA is characterized by apneas and hypopneas, which are caused by partial or complete collapse of the upper airways. According to ICSD-3, an obstructive apnea is diagnosed when the breathing disorder cannot be explained by any other sleep disorder or medical condition, or by the use of drugs or other substances, and an apnea–hypopnea index (AHI) >15/h (each event ≥10 s) sleep time or an AHI ≥5/h sleep time in combination with a typical clinical pathology or relevant comorbidity is present.

Clinical pathology: Daytime sleepiness up to the extent of involuntarily falling asleep is the main clinical symptom of OSA. However, some affected patients exhibit no sleepiness, do not consider it to be a symptom of disease or do not explicitly notice it. Daytime sleepiness reduces productivity and, during the course of disease, also impairs cognitive ability, social compatibility, and quality of life, for example. Sleeping partners report breathing arrests. The most important diagnostic parameter is the AHI, which reports the number of apneas and hypopneas per hour of sleep. The AHI objectifies the diagnosis and, together with the clinical symptoms and comorbidities, determines the severity of OSA. An AHI >15/h and <30/h is defined as moderate, an AHI >30/h as severe OSA.

### 2.2 Central sleep apnea

This group of sleep-related breathing disorders is characterized by dysregulation of respiratory control mechanisms and/or transfer of impulses to the thoracic skeleton. In central apnea, despite open or passively collapsed upper airways, there is no respiratory airflow and, consequently, effective ventilation does not occur. For the entire duration of the suspended airflow, there is no inspiratory respiratory effort. In central hypopneas, respiratory effort and airflow are reduced. In contrast to OSA, there are no signs of paradoxical breathing.

Central sleep apnea is subdivided into hypercapnic and non-hypercapnic forms. Hypercapnic breathing disorders are characterized by reduced respiratory drive, or transfer to or action upon the muscles of respiration (e. g., neuromuscular diseases). In non-hypercapnic forms of central apnea, respiratory drive is usually increased and/or increased chemosensitivity is present (e. g., central sleep apnea at high altitude, central sleep apnea with or without Cheyne–Stokes breathing in cardio-/cerebrovascular diseases and renal insufficiency).

### 2.3 Sleep-related hypoventilation/sleep-related hypoxemia

In contrast to ICSD-2, ICSD-3 differentiates between sleep-related hypoventilation and sleep-related hypoxemia. Sleep-related hypoventilation is differentiated into six separate entities, whereas no subclassification is suggested for sleep-related hypoxemia. According to ICSD-3, *sleep-related hypoxemia *is present when *polysomnography or nocturnal pulse oximetry* documents an *oxygen saturation ≤88% lasting for at least 5 min* in the absence of sleep-related hypoventilation, i. e., no hypercapnia is present. Sleep-related hypoxemia is usually a result of a general medical or neurological disease, and cannot be explained by a sleep-related breathing disorder alone. Some patients with sleep-related hypoxemia also exhibit hypoxemia during the day.

## 3. Diagnosis

Diagnosis of sleep-related breathing disorders is a prerequisite to initiation of efficient, target-oriented, economic treatments with few side effects. The diagnostic instruments are oriented to the pathophysiology, consequences, and comorbidities of sleep-related breathing disorders. They serve to define the severity of the disorder and a patient’s comorbidities, and should be able to estimate the extent of the consequences. They include recording of case history, self-rating questionnaires, in- and outpatient multichannel devices, video recording, and clinical laboratory diagnostic tests, as well as instrument-based and non-instrument-based performance tests (Tables 2 and 3). Alone or in combination, all of the employed diagnostic instruments serve to establish diagnosis and monitor the effects of treatment. Beyond this, they are necessary for sociomedical evaluation and assessment.

**Table 2:** Diagnostic instruments for the different categories of sleep-related breathing disorders

	Questionnaires	Performance and vigilance tests	1–3-channel polygraphy	4–6-channel polygraphy	Polysomnography
Obstructive sleep apnea	(+)	(+)	(+)	+	+
Central sleep apnea	(+)		(+)	(+)	+
Sleep-related hypoventilation				(+)	+
Sleep-related hypoxemia				(+)	+

*+* use recommended, *(+)* use possible under particular conditions, *no entry* the method is neither recommended nor rejected, i. e., there is no evidence for the method or it is not possible or it is economically nonviable

**Table 3:** Questionnaires and instruments used for vigilance diagnostics. The validated instruments assess complaints, impairments to wellbeing, symptoms, and various behavioral patterns

	ESS	Berlin Q	STOP-BANG	Waist to height	PVT, Osler, DASS	MSLT/MWT
Obstructive sleep apnea	(+)	(+)	(+)	(+)	(+)	(+)
Central sleep apnea		(+)				(+)
Sleep-related hypoventilation						(+)
Sleep-related hypoxemia					(+)	

*+* use recommended, *(+)* use possible under particular conditions, *no entry* the method is neither recommended nor rejected, i. e., there is no evidence for the method or it is not possible or it is economically nonviable

Depending on the particular case scenario, the diagnostic methods may be applied in combination, simultaneously or sequentially, complementarily or exclusionarily, with different demands in terms of time, personnel, organization, and materials.

The most important diagnostic instrument and the gold standard reference for sleep medicine diagnostics in the sleep laboratory is supervised cardiorespiratory polysomnography (PSG; Table 4). Recording and evaluation of the PSG should be performed according to the AASM criteria (version 2.3).

**Table 4:** Recommended channels for cardiorespiratory polysomnography. The table presents the functions to be assessed, the corresponding biosignal, the technical requirements, and the technical specifications in terms of optimal sampling rate and filter settings

Function	Parameter	Technical requirements	Optimal sampling rate	Filter
Sleep	EEG, EOG	Electrodes	500 Hz	0.3–35 Hz
	EMG	Electrodes	500 Hz	10–100 Hz
Breathing	Oronasal airflow	Nasal pressure cannula, thermistor	100 Hz	0.1–15 Hz
	Respiratory effort	Induction plethysmography	100 Hz	0.1–15 Hz
	Oxygen saturation	SaO_2_	25 Hz	–
	Carbon dioxide saturation	tcPaCO_2_	25 Hz	–
	Snoring	Microphone	500 Hz	–
Cardiac	ECG	Electrodes	500 Hz	0.3–70 Hz
Movement	EMG tibialis muscle	Electrodes	500 Hz	10–100 Hz
	Body position	Position sensor	1 Hz	–
	Video	Video camera	5 Hz	–

*EEG* electroencephalogram, *EOG* electrooculogram, *EMG* electromyogram, *ECG* electrocardiogram, *SaO*_*2*_ oxygen saturation, *tcPaCO*_*2*_ transcutaneous measurement of carbon dioxide partial pressure

For diagnosis of sleep-related breathing disorders, simplified portable systems are available. Using 4–6 channels, these can measure breathing-related parameters without recording sleep electroencephalograms (EEGs).

### 3.1 Diagnostic recommendations

The sleep-related breathing disorders algorithms (Figs. 1, 2, 3 and 4) and the decision paths represent a guide to selection of particular diagnostic instruments.

#### Non-instrument-based diagnostics

- For evaluation of the nasal structures relevant to airflow, clinical examination of the nose should be performed, which may also include endoscopy (C).
- Examination of the oral cavity and pharynx is highly relevant and should be performed (B).
- If therapy with a mandibular advancement device (MAD) is considered, the possible mandibular protrusion and dental status should be assessed, which can be complemented by a panoramic radiogram (PSA, panoramic x ray; B).
- The diagnostic workup of OSA should include evaluation of facial skeleton morphology for orientation (B). This may include a lateral cephalometric radiograph (Lat Ceph) in order to assess, e. g., the posterior airway space (PAS).
- To detect skeletal abnormalities, a cephalometric radiograph may be recommended. The PAS should be measured extending from the inferior border of the mandible. For distances below 10 mm, a narrowed airway can be assumed. Further confirmation might be obtained from three-dimensional imaging of the upper airway or by performing transnasal videoendoscopy. Adequate dentition is a prerequisite for modeling of a MAD, with at least eight loadable teeth or equivalent implants in the maxilla and mandible or an equivalent implant supply. In this scenario, a panoramic radiogram should be made and evaluated by a dentist experienced in sleep medicine (B).
- For differential diagnosis of the causes of OSA, individual patients should be offered dental and specialist radiographic examination by qualified sleep medicine dentists, orthodontists, or oral and maxillofacial surgeons, including cephalometric radiography to investigate the possibility of treatment with a MAD or corrective jaw osteotomy.

#### Instrument-based diagnostics

- After assessing the abovementioned pretest probability, instrument-based diagnostics can be divided into the following three categories: preliminary diagnostics, confirmatory diagnostics, differential diagnostics (C).

##### Polysomnography

- PSG in the sleep laboratory monitored and supervised by qualified sleep medicine personnel is recommended as the gold standard and reference method (A).
- PSG should be performed according to current guidelines. This includes recording of sleep EEG, EOG, EMG, and ECG; oronasal airflow; snoring; respiratory effort; oxygen saturation; body position; and a video (A; Table 4).
- Videometric analysis should be performed for diagnosis of parasomnias and sleep movement disorders, and for differential diagnostic discrimination from several types of epilepsy (A).
- In cases with a low pretest probability or suspicion of sleep diseases other than OSA by medical history, PSG is indicated for the differential diagnosis (A).

##### Polygraphy for sleep-related breathing disorders

- Polygraphy systems with a reduced number of channels can be used provided they include at least one measurement of oxygen saturation, oronasal airflow, respiratory effort, heart or pulse rate, and body position (A). Such systems should only be used when there is a high pretest probability of obtaining diagnostic confirmation or for assessment of the severity of sleep-related breathing disorders (A).
- Polygraphy systems for diagnosis of sleep-related breathing disorders should be applied by specialized sleep medicine physicians, who can assess and evaluate the pretest probability, the symptoms, and the comorbidities (A).
- In general, polygraphy should not be used as a substitute for PSG for the diagnosis of sleep-related breathing disorders in patients with relevant comorbidities (A). The recorded signals should be scored visually by trained personnel. Evaluation by so-called automatic scoring alone is currently not recommended (A).
- For diagnosis of sleep-related breathing disorders by exclusion, cardiorespiratory PSG is recommended; polygraphy is not sufficient (A).
- Neither polygraphy nor PSG is sufficient to clarify hypercapnic respiratory failure (A).
- Disease course and treatment can be monitored polygraphically. In patients with an unclear response to therapy, patients with a high cardiovascular risk, and patients with other sleep-impairing diseases, PSG monitoring may be necessary (C).

##### Reduced monitoring for sleep-related breathing disorders

- Polygrams fulfilling fewer than all of the abovementioned criteria can give an indication of the existence of a sleep-related breathing disorder and increase the pretest probability. Polygraphy is not, however, recommended as a stand-alone method for diagnosis of sleep-related breathing disorders (A).
- In the presence of cardiovascular diseases that increase the risk of sleep-related breathing disorders (arterial hypertension, heart failure, atrial fibrillation, cerebrovascular disease) and the absence of all typical symptoms, a single- or two-channel registration is possible. If this registration supports the presence of OSA, further diagnostic workup with polygraphy or PSG is indicated (C).

## 4. Obstructive sleep apnea—recommendations

The algorithm for treatment of patients with OSA is shown in Fig. 3. Clinical guidelines for manual CPAP-titration are given in Table 5.

### 4.1 Positive airway pressure therapies

- Continuous positive airway pressure (CPAP) therapy is the gold standard for treatment of OSA syndrome (A).
- CPAP therapy should be administered for moderate and severe sleep apnea (AHI >15/h; A).
- In cases of mild sleep apnea (AHI ≤15/h) with a cardiovascular risk and/or daytime fatigue, CPAP therapy can be considered (C).
- For initial titration, structured patient training should take place (B).
- Therapeutic decisions regarding the CPAP treatment mode should be made by a physician qualified in sleep medicine (A).
- Minimal time should elapse between titration of the respiratory therapy and provision of a therapeutic device (B).
- It is recommended that the selection of the device, mask, and additional supportive aids, as well as the initial fitting, be performed by personnel qualified in sleep medicine (C).
- Initiation of CPAP therapy or a modified positive airway pressure treatment should be performed in a sleep lab with polysomnographic monitoring (A).
- The final adjustments should be performed with the same mask and device that the patient will receive for home use.
- Whenever clinically possible, bilevel therapy should always be preceded by a therapeutic trial with CPAP or automatic CPAP (APAP) (B).
- APAP and CPAP are of equal value for titration of treatment and long-term therapy of OSA (A).
- APAP should not be used to treat in central sleep apnea or nocturnal hypoventilation (B).
- The first therapeutic monitoring should occur within the first 6 weeks and be based on clinical examination; additional polygraphy should be performed with at least 6 channels. Subsequent treatment should be monitored at least annually (B).
- Polygraphic or polysomnographic monitoring should be performed in patients with subjective complaints, or clinical or technical problems (A).

The clinical guidelines for manual CPAP titration are summarized in Table 5.

#### CPAP-intolerant patients

- For patients, who cannot be treated with CPAP, other types of respiratory support or other suitable treatments should be employed (A).

#### Patients with dementia

- In patients with mild or moderate dementia and OSA, an attempt of CPAP treatment should be made.

**Table 5:** Clinical guidelines for manual CPAP titration

1. Adequate patient information, instruction, and adaptation of therapy
2. Titration of the CPAP pressure at which apneas, hypopneas, RERAs, and snoring no longer occur
3. Commence titration with 4 mbar (CPAP), or IPAP 8/EPAP 4 mbar (bilevel)
4. Max. CPAP: 15 mbar, max. IPAP: 20 mbar (bilevel), IPAP/EPAP difference: min. 4, max. 10 mbar
5. Where necessary, increase the pressure in 1‑mbar increments with a time interval of at least 5 min
6. Pressure is increased when at least two OSAs or three hypopneas or five RERAs or 3 min of loud snoring occur
7. Change to bilevel in cases of intolerance to CPAP or pressure >15 mbar
8. Therapeutic goal: RDI <5/h, min. oxygen saturation >90%
9. Optimal titration: RDI <5/h for at least 15 min, including REM sleep and no arousals
10. Good titration: RDI ≤10/h or 50% reduction of baseline in cases with a pretreatment RDI <15/h, including occurrence of REM sleep and reduction of waking reactions
11. Adequate titration: RDI >10/h, but a reduction by 75% of the baseline value; particularly in patients with severe OSAS or in patients with optimal titration in whom no REM sleep occurs
12. Unacceptable titration: fulfills none of the abovementioned criteria; and
13. A second titration night is necessary when the criteria for optimal/good titration are not fulfilled during the first night

*CPAP* continuous positive airway pressure, *IPAP* inspiratory positive airway pressure, *EPAP* expiratory positive airway pressure,* RERA* respiratory effort related arousal, *RDI* respiratory disturbance index, *REM* rapid eye movement, *OSA* obstructive sleep apnea, *OSAS* OSA syndrome

### 4.2 Non-positive airway pressure therapies

#### Weight reduction

- Weight reduction strategies should be recommended as supportive therapy to all overweight patients (A).

#### Mandibular advancement devices

- MADs may be an alternative to positive airway pressure therapy in patients with mild to moderate OSA (AHI ≤30/h). This is particularly relevant for patients with a body mass index (BMI) < 30 kg/m^2^ and a lifetime history of sleep apnea (A).
- In patients with a high AHI and/or BMI >30 kg/m^2^, a MAD can be considered where positive airway pressure therapy cannot be used despite use of all supportive measures (C).
- MADs should be fitted by personnel with dental and sleep medicine expertise (A).
- The effect of MAD therapy should be regularly monitored, e.g., annually, by physicians qualified in sleep medicine (A).

#### Drug-based treatments

- Drug treatment of OSA cannot be recommended (A).
- Modafinil (off-label) treatment can be considered for residual daytime sleepiness due to OSA in patients on CPAP therapy, provided other causes have been excluded (C).

#### Oxygen therapy

- Nocturnal oxygen therapy should not be used as the sole treatment for OSA (A).

#### Therapies to increase muscle tone

- Surface electrical stimulation to increase muscle tone should not be performed (B).
- Non-electrical methods and myofunctional exercises can be considered in individual cases (B).

#### Positional therapy

- For patients with mild to moderate position-dependent OSA, therapy to prevent sleeping in the supine position can be considered when no other treatments recommended in this guideline is applicable or where these are not adequately tolerated (C).

#### Surgical interventions

- Surgery to improve nasal breathing should be considered in patients with impaired nasal breathing resulting in CPAP intolerance (B).
- In patients with tonsillar hypertrophy and oropharyngeal obstruction, tonsillectomy should be performed, particularly when an alternative therapy (CPAP, MAD) is not possible or not sufficiently tolerated (A). Tonsillectomy may be combined with uvulopalatopharyngoplasty (C).
- In the absence of anatomic abnormalities, neurostimulation of the hypoglossal nerve can be used in patients with moderate to severe OSA when positive airway pressure therapy meeting the abovenamed criteria cannot be employed. Neurostimulation should only be used in case of CPAP intolerance or ineffectiveness with AHI 15–50/h and ≤class I obesity, provided no concentric obstruction is documented in sleep endoscopy (B).
- In the presence of appropriate anatomic findings with a small mandible and narrow facial skeleton (PAS <10 mm on a cephalometric radiograph), advancement of the maxilla and/or mandible (bimaxillary/maxillomandibular advancement) should be considered, particularly when an alternative therapy (CPAP, MAD) is not possible or is insufficiently tolerated (A).
- Muscle-resecting surgery to the soft palate is not recommended (A).
- Several other surgical interventions may be expedient, depending on the anatomic findings (C).

## 5. Perioperative management of patients with obstructive sleep apnea—recommendations

- Questions addressing OSA should be included in preoperative assessments of medical history (B).
- In case of suspected undiagnosed OSA a diagnostic sleep work-up should be performed; however, the priority of the surgical procedure must be weighed against the necessity/type of sleep medicine diagnostic workup on an individual basis (B).
- In the presence of OSA requiring treatment, a previously titrated CPAP therapy should be continued in the perioperative phase; in the absence of a previously titrated CPAP therapy, initiation CPAP treatment should be considered, provided permissible by the priority of surgery (B).
- The selection of the type of anesthesia, as well as the type and duration of potentially necessary postoperative monitoring, should be oriented toward the type and the seriousness of surgery, the perioperative analgesic requirements, the severity of the (assumed) breathing disorder, and the patient’s individual risk constellation, including sleep apnea-associated comorbidities (B).

## 6. Diagnosis and treatment of central sleep apnea—recommendations

The algorithm for management of patients with suspected central sleep apnea is shown in Fig. 4.

### Diagnosis

- When diagnosis of central sleep apnea has been confirmed, possible general medical, pharmacological, and neurological causes should be investigated (A).

### Treatment of central sleep apnea in patients with heart failure with preserved left ventricular ejection fraction (HFpEF)

- In HFpEF patients (left ventricular ejection fraction, LVEF, >45%), CPAP therapy or adaptive servoventilation (ASV) should be used to treat central sleep apnea (B).
- In HFpEF patients (LVEF >45%), oxygen therapy can be used to treat symptomatic central sleep apnea where CPAP or, in the presence of the correct indication, ASV therapy has failed (C).

### Treatment of central sleep apnea in patients with heart failure with reduced left ventricular ejection fraction (HFrEF)

- For treatment of central sleep apnea in HFrEF patients, the heart failure should be treated according to current guidelines (A).
- In patients with moderate to severe central sleep apnea and symptomatic HFrEF (LVEF ≤45%), ASV therapy should not be performed (A).
- In selected patients with symptomatic moderate to severe central sleep apnea and HFrEF (LVEF ≤45%), therapy with CPAP can be considered (C).
- In patients with symptomatic moderate to severe central sleep apnea and HFrEF (LVEF ≤45%), therapies for which there are no randomized long-term studies, e. g., unilateral stimulation of the phrenic nerve and oxygen therapy, should only be applied within prospective studies (B).

### Treatment of central sleep apnea in patients with heart failure

- Alternative therapies such as bilevel in spontaneous time (ST) modus, acetazolamide, or theophylline should not be used to treat normo- or hypocapnic central sleep apnea in patients with heart failure (B).

### Recommendations for central sleep apnea due to periodic breathing at high altitude

- Acetazolamide can be recommended in healthy individuals to reduce central apneas/periodic breathing at high altitude and to improve nocturnal oxygen saturation at high altitude (C).
- Combined therapy comprising acetazolamide and CPAP can be recommended to prevent worsening of central sleep apnea/periodic breathing at high altitude in patients with previously diagnosed sleep-related breathing disorders (C).

### Recommendations for central sleep apnea due to medications, drugs, or substances

- In opioid-induced sleep apnea a reduction in opioid dosage should be considered (B).
- Positive airway pressure therapies should be applied on an individual basis in opioid-induced sleep apnea, and their efficiency should be assessed polysomnographically (A).
- In individual cases, positive airway pressure therapies and oxygen administration may be combined (C). Therapy initiation and monitoring should include capnography in addition to polysomnography (A).

### Recommendations for idiopathic central sleep apnea

- Treatment can be performed with noninvasive ventilation therapy or as spontaneous breathing with a background frequency (C).

### Recommendations for central sleep apnea due to therapy

- Known triggers (e. g., over-treatment or split nights) should be eliminated (A).
- In patients with central sleep apnea due to therapy who require treatment and who are normo- or hypocapnic, therapy should be switched to ASV (B).

## 7. Diagnosis and treatment of sleep-related hypoventilation/sleep-related hypoxemia

**Table 6:** Diagnostic criteria of obesity hypoventilation syndrome

a. Hypercapnia (daytime PaCO_2_ >45 mmHg)
b. Body mass index >30 kg/m^2^
c. Hypoventilation is not primarily caused by another disease

### Diagnostic recommendations

- In the presence of clinical indications of sleep-related hypoventilation or predisposing primary disease, diagnosis should be performed via nocturnal arterial or capillary blood gas analysis, or nocturnal measurement of transcutaneous or end-tidal CO_2_. For diagnosis of obesity hypoventilation syndrome, a daytime blood gas analysis is required (Table 6). Sleep-related hypoxemia should be diagnosed by nocturnal oximetry in combination with measurement of CO_2_ during the night (A).
- In patients with BMI >30 kg/m^2^ and symptoms of a sleep-related breathing disorder, the awake-state venous bicarbonate, or arterial or capillary pCO_2_, or transcutaneous or end-tidal CO_2_ should be measured in order to exclude the simultaneous presence of sleep-related hypoventilation (Table 6; A).
- Transcutaneous capnometry is recommended as the most sensitive method for demonstrating the presence of sleep-related hypercapnia. This can be performed in combination with polygraphy or PSG (C).
- In patients with neuromuscular or chest wall diseases, sleep-related hypoventilation should be ruled out in candidates for ventilation therapy with a vital capacity <50% (A).
- PSG is the gold standard for diagnosis of exclusion and differential diagnosis of sleep-related breathing disorders in the context of sleep-related hypoventilation or hypoxemia (A).

### Treatment

- In patients with obesity hypoventilation, an attempt at CPAP therapy with CO_2_ monitoring can be undertaken (C).
- If nocturnal hypoventilation persists under CPAP therapy, noninvasive pressure-support ventilation (without or with target volume) should be initiated (B).
- Oxygen therapy alone is not recommended in patients with obesity hypoventilation syndrome (A).
- After utilisation of all other weight-reduction strategies in patients with obesity hypoventilation syndrome, bariatric surgery can be considered (B).

### Indications for initiation of noninvasive ventilation

- In patients with symptomatic obstruction of the lower airways, or neuromuscular or chest wall diseases with hypercapnia in the awake-state (PaCO_2_ ≥50 mmHg for diseases with obstruction of the lower airways or ≥45 mmHg in neuromuscular or chest wall diseases) or while asleep (PaCO_2_ ≥55 mmHg or ≥50 mmHg, respectively, or ∆PtcCO_2_ ≥10 mmHg compared normocapnic awake-state), initiation of noninvasive ventilation is recommended (Table 7; A).

**Table 7:** Indications for initiation of noninvasive ventilation

**Obesity hypoventilation syndrome**
– Nocturnal transcutaneously measured CO_2_ level under CPAP therapy >55 mmHg for at least 5 min
or
– Nocturnal oxygen saturation <90% for at least 10 min
**Diseases with obstruction of the lower airways**
– Hypercapnia while awake (*PaCO*_*2*_ * ≥50 mmHg*)
or
– Hypercapnia while asleep (PaCO_2_ ≥55 mmHg or ∆PtcCO_2_ ≥10 mmHg compared to normocapnic awake-state)
**Neuromuscular or chest wall diseases**
– Hypercapnia while awake (*PaCO*_*2*_ * ≥45 mmHg*)
or
– Hypercapnia while asleep (PaCO_2_ ≥50 mmHg or ∆PtcCO_2_ ≥10 mmHg compared to normocapnic awake-state)

